# Is Herd Immunity Against SARS-CoV-2 a Silver Lining?

**DOI:** 10.3389/fimmu.2020.586781

**Published:** 2020-09-30

**Authors:** Ramachandran Vignesh, Esaki M. Shankar, Vijayakumar Velu, Sadras Panchatcharam Thyagarajan

**Affiliations:** ^1^ Preclinical Department, Royal College of Medicine Perak (UniKL RCMP), Universiti Kuala Lumpur, Ipoh, Malaysia; ^2^ Infectious Diseases Laboratory, YRG Centre for AIDS Research and Education, Chennai, India; ^3^ Infection Biology, Department of Life Sciences, Central University of Tamil Nadu, Thiruvarur, India; ^4^ Division of Microbiology and Immunology, Yerkes National Primate Research Center, Emory University, Atlanta, GA, United States; ^5^ Department of Pathology and Laboratory Medicine, Emory Vaccine Center, Emory University, Atlanta, GA, United States; ^6^ Central Research Facility, Sri Ramachandra Institute of Higher Education and Research, Chennai, India

**Keywords:** herd immunity, coronavirus disease 2019, severe acute respiratory syndrome coronavirus-2, seroprevalence, vaccines

## Introduction

The emergence of a novel coronavirus severe acute respiratory syndrome coronavirus-2 (SARS-CoV-2) in late 2019 and its wide global spread has led to millions of infections and substantial morbidity and mortality (1). Coronavirus disease 2019 (COVID-19) caused by SARS-CoV-2 infection can range from mild self-limiting disease to acute respiratory distress syndrome and death (2). With the WHO having reported 31,174,627 confirmed cases and 962,613 deaths globally as of 22nd September 2020, the COVID-19 pandemic seems to show almost poor indication of abating (3). While the global scientific community is racing against time to strategize combating possibilities, with several vaccine trials, drug discoveries and validations underway, we still need a practical and sustainable solution to face the ongoing threat to global public health.

The terminology “herd immunity”, coined by Topley and Wilson in 1923, later formed the basis for vaccines, applications and vaccination programs, especially against certain viral infections (4). The concept of herd immunity refers to the indirect protection from infection conferred on susceptible individuals when a sufficiently large proportion of individuals immune to the infection exist in a population. Herd immunity concept is generally applicable to those infections that are transmitted directly from person to person and for those humans serving as the reservoir of infection. History has shown that a significant drop in the number of cases and even eradication is rendered by vaccines, and herd immunity is achieved against infectious diseases like small pox, polio, measles, rubella, diphtheria, pertussis and mumps (4–7). The concept of herd immunity appears to be highly critical in the context of disease elimination programs because the said eradication of an infectious agent becomes possible in spite of the absence of an effectual vaccine. Interestingly, natural herd immunity is a classical concept that has been successfully accomplished in instances like the 1918 H1N1 influenza pandemic wherein no vaccine was available (8).

Going by this old school modus operandi, in the absence of a vaccine, building natural herd immunity against SARS-CoV-2 is theoretically considered feasible. However, letting an existing super infectious condition to run amok in the pretext of building up effective herd immunity might not be a smart strategy to end the pandemic. It requires judicious analyses to avoid the colossal burden it could inflict on the healthcare system and the surge in mortalities and associated complications.

In a detailed classical analysis, Fox et al. has listed down the following four conditions for effective herd immunity to ensue (i) the infectious agent must exist and be restricted to a single host, (ii) the transmission must occur primarily through direct contact, (iii) the infection must induce a robust and life-long immunity, and (iv) the cumulative or herd immunity is amplified if the population possesses a random mixing pattern (9). Applying the aforementioned conditions to herd immunity against SARS-CoV-2, though the infectious agent has been identified and believed to be zoonotic, we are yet to place a finger on the intermediate host (10). Secondly, the transmission, of course, occurs through direct person-to-person contact (11). However, regarding the third condition, we have a paucity of data on the immune response elicited by SARS-CoV-2 in humans, till date (12) and the questions remains about the long-lasting immunity for the exposed individuals. Finally, though the entire human population is susceptible to COVID-19, the mixing of the pattern varies that is dependent on several societal factors and practices such as universal lockdown, mass quarantine, isolation, social distancing and public health preventive measures, particularly for those at risk (12).

**Table 1** listed the various infectious agents and their corresponding R_0_ values and herd immunity thresholds. While earlier studies have estimated the basic reproductive number (R_0_) of SARS-CoV-2 to be in the range of 2 to 3, recent estimates place the R_0_ at 5.7 (13, 15). This indicates the highly infective nature of the virus, meaning that on an average each infected individual can give rise to about 5.7 newer infections and subsequently spread the agent on an exponential scale. Assuming an R_0_ estimate of 5.7, using the mathematical formula 1-1/R_0_, the herd immunity threshold for COVID-19 would be ~82.5%, meaning that the incidence of infection will begin to decline once the proportion of individuals with acquired immunity to SARS-CoV-2 in the population exceeds 82.5%. However, it remains to be noted that the estimate of the proposed threshold is only theoretical with the assumptions of a homogenous population and presence of uniform sterilizing immunity in the recovered patients. Mathematical modeling studies have shown that disease-induced herd immunity threshold would be substantially lower than the values calculated by conventional formula due to the population heterogeneity (16, 17).

**Table 1:** R_0_ values and corresponding herd immunity threshold values of infectious diseases (5, 6, 13, 14).

S. no.	Infectious diseases	R_0_ value	Herd immunity threshold
1	Small pox	5–7	80–85%
2	Mumps	4–7	75–86%
3	Measles	12–18	92–94%
4	Diphtheria	6–7	85%
5	Pertussis	12–17	92–94%
6	Polio	4–13	75–92%
7	Rubella	6–7	83–85%
8	H1N1 (2009 Pandemic)	1.6	40%
9	SARS	2–4	50–75%
10	SARS-CoV-2 (COVID-19)	5.7	82.5%

Furthermore, there are several instances of “super spreading events” reported from various countries, wherein a single patient goes on to infect far more number of people than an average patient does, based on estimated R_0_ value (18). Several factors such as increased viral load, asymptomatic individuals, immune suppression and extensive social interactions have been implicated in these “super-spreading events” (19). Studies point towards these “super-spreaders” as the reason for the heterogeneous propagation of SARS-CoV-2 across geographical locations and since these present with a relatively higher R_0_ value, they could potentially impact the dynamics of spread and lower the herd immunity threshold (20, 21). The whole concept of herd immunity against SARS-CoV-2 hinges on the assumption that an infection would generate sufficient, effective and protective long-lasting immunity. However, data are scarce if the acquired immunity developed by humans is sterilizing enough and whether it would stay long enough.

## Rays of Hope

Earlier studies on survivors of SARS-CoV-1 and MERS-CoV infections have shown that the antibodies against SARS-CoV-1 persisted for nearly two years and antibodies to MERS-CoV lasted for almost 3 years (22, 23). Besides, studies have also demonstrated evidence of seroconversion within 14 to 19 days of disease onset among SARS-CoV-2 patients with COVID-19 (24, 25). A study has reported the interesting finding of T cell reactivity to SARS-CoV-2 in about 50% of specimens collected between 2015 and 2018 before the viral emergence (26). This could reflect the circulating T cell memory to the seasonal coronaviruses- 229E, OC43, NL63, and HKU1. A study also suggests that SARS-CoV-2-reactive antibody responses have been detected in unexposed individuals who are IgG seropositive for OC43 and NL63 and this cross-immune reactivity mainly targets viral 1AB polyprotein and S proteins, these viral antigens have high sequence similarity with low pathogenic human coronaviruses and SARS-CoV-2 (27, 28). In addition, a study has also shown that these antibodies were particularly prevalent in children and adolescents (29). It is also important to note that sera from uninfected individuals exhibited variability in their reactivity, they are reactive with SARS-CoV-2 S and nucleoprotein but not with the S1 subunit or the receptor binding domain (RBD) of spike protein. Since many studies from different geographical locations are documenting preexisting immunity to SARS-CoV-2, it will be important to define specificities of these T and B cell immune response carefully to assess their association with COVID-19 disease severity. This preexisting cross-reactive T and B cell immunity to SARS-CoV-2 may have wide implications as this could explain differential clinical outcomes in COVID-19 patients, disease severity, vaccine development, and important in accessing herd immunity for SARS-CoV-2 viral infection/COVID-19 disease.

Studies characterizing the SARS-CoV-2-specific T cell responses suggest that there is marked activation of T cells in acute COVID-19 patients (30–32). Several studies have provided strong evidence for the importance of SARS-CoV-2 specific CTLs, and T helper cells in mild and moderate patients compared to severe COVID-19 disease (27, 28, 31–33). The effector memory CD8 T cell population is decreased in the severe patients compared to the recovered patients (34–36). Similarly, the T cells in severe patients compared to convalescent patients express higher levels of exhaustion markers PD-1 and CD39 on the memory CD8 T cells with the signature pertaining to hyperimmune activation (HLADR^+^CD38^+^CD8^+^) with lower CD8 T cell response. In line with CD8 T cells, the effector memory CD4 T cell frequencies are also elevated in the COVID-19 patients (34–36). Interestingly, the recovered patients seem to have higher levels of activated circulating T follicular helper (Tfh) population, indicative of recent antigen encounter and emigration from germinal center. Similar to the PD-1 levels of CD8 T cells, the PD-1 levels were increased in the CD4 T cells. As a result of the decrease in the T cells, non-T cell frequencies that includes, macrophages, monocytes, neutrophils were observed to be elevated in severe COVID-19 patients (30, 34). Similar to T cells the B cell subpopulations are altered in the COVID-19 disease and it has been shown that the frequency of plasmablast is highly elevated (30% of the circulating B cells) in the COVID-19 patients (34). These blood plasmablast frequencies are shown to correlate with activated circulating Tfh cells (34, 37). The proliferating B cells are markedly elevated in the COVID-19 patients with activated phenotype, suggestive of altered B cell response during COVID-19 disease. Several studies have provided strong evidence for the importance of SARS-CoV-2 - specific neutralizing antibodies in association with less disease severity in COVID-19 patients (38, 39). SARS-CoV-2 specific antibodies are found in convalescent plasma and most recovered individuals have RBD-specific antibodies with potent antiviral activity (40, 41), importantly severe and critical patients are currently being treated with convalescent plasma therapy in many parts of the world (42, 43). Overall, these data suggest that vaccines or therapeutic strategies that induce functional anti-viral SARS-CoV-2 specific T and B cell responses are important to curtail SARS-CoV-2 infection *in vivo*.

## Enigma of Unknowns

There remain many unknowns and several other research questions unanswered. The degree of protection afforded by the antibodies against SARS-CoV-2 among the infected is still ambiguous, and strong evidence-based findings are awaited to know if the host responses generated would be of strong sterilizing or weak and waning immune responses. Earlier studies on the coronavirus 229E, responsible for common cold have shown that the titers of antibodies produced were not sufficient to prevent reinfection in all the individuals studied (44). Interestingly, similar pattern exists in SARS-CoV-2 infection and where there is a rapid decay of SARS-CoV-2 of antibodies in persons with mild symptoms and recovered patients (45, 46). Similarly, as a recent study demonstrated the evidence of reinfection of SARS-CoV-2 by phylogenetically distinct SARS-CoV-2 re-infection, this may also suggest that the immunity generated during primary SARS-CoV-2 infection may be short lived and may not protect if reinfection happens by the second distinct virus (47). These results also suggest that SARS-CoV-2 may continue to circulate among human population despite herd immunity with natural infection or with vaccination. However, a recent study on monkeys has indicated the development of protective immunity against re-exposure to SARS-CoV-2 (homologous) (48). Hence, massive longitudinal monitoring of SARS-CoV-2 seroprevalence remains the need of the hour to determine the percentage of the population already infected and if reaching a herd immunity threshold is even feasible. It also remains logical to consider the viral factors such as the possibility of mutations and emergence of new strains of a virus, which can go on to make herd immunity futile (49).

## Potential Consequences and Way Forward

**Figure 1:**
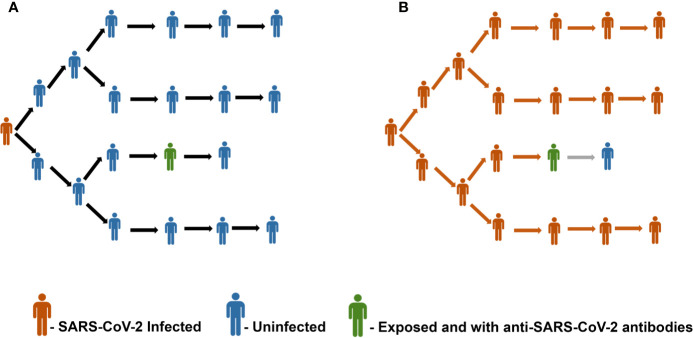
**(A)** Representative image of a scenario with a population having 5% seroprevalence of antibodies against SARS-CoV-2. Panel **(B)** depicts the unchecked spread of infection due to the insufficient herd immunity.

Given the unavailability of a vaccine against SARS-CoV-2, it is prudent to foresee the consequences of achieving the herd immunity threshold in epidemiological and immunological viewpoints. A recent population-based sero-epidemiological study from Spain involving 61075 participants has revealed ~5% seroprevalence of SARS-CoV-2, thereby highlighting that the majority of the Spanish population remains seronegative (50). **Figure 1** represents this scenario of a population with only 5% seroprevalence of SARS-CoV-2 antibodies and the importance of achieving herd immunity threshold. Similarly, seroprevalence among the community in Los Angeles County has been reported as 4.34%, about 3.3% in Japan, and 2.73% in Hong Kong (51–53). Based on a serological survey conducted by the Indian Council for Medical Research (ICMR) in May - June, 2020 across 83 districts in India involving over 26,400 participants, the seroprevalence was found to be only 0.73% (54). The lower rates of seroprevalence of SARS-CoV-2 antibodies reported from various geographical locations point in the direction that we are still a long way ahead of achieving herd immunity.

According to a modeling study, assuming a scenario of achieving uniform herd immunity threshold of 67% and with an infection fatality rate of 0.6% for SARS-CoV-2, the estimate of the absolute number of deaths worldwide would easily exceed a whopping 30 million (14). Since these estimates are based on various assumptions and the real-life scenario could throw us curve balls of multiple factors influencing the outcomes, the fatality rate could even be higher. A recent modelling study has estimated that about one in five individuals worldwide would be at increased risk of severe COVID-19, upon infection with SARS-CoV-2, owing to the underlying conditions. The study projects an alarming figure of about 349 million people requiring hospital admission and substantial mortality rates (55). Furthermore, in the case of highly populated and resource-strapped countries, the increase in the number of infected cases could overwhelm the healthcare facilities and could lead to a shortage of essential medical services exacerbating further complications and deaths.

## Conclusions

Thus, weighing on the immunological and epidemiological consequences, achieving natural herd immunity at the cost of severe morbidities and mortalities worldwide, in the absence of an effective and safe vaccine appears farfetched and seemingly an impracticable solution. With so many vaccines being tested in various phases of clinical trials, the availability of an effective vaccine seems to be the only way forward in this war against COVID-19. Echoing Dr. Anthony Fauci’s statements, even when a vaccine with a modest efficacy is made available in near future, anti-science and anti-vaccine campaigns need to be counteracted and cooperation of the general public is needed to achieve an efficient and successful herd immunity against COVID-19 (56).

## Author Contributions

RV, ES, VV, and ST led the writing of this opinion article. All authors contributed to the article and approved the submitted version.

## Funding

VV was supported by Emory University CFAR grant P30 AI050409 and NCRR/NIH base grants P30 RR00165, P51OD011132 (to Y.N.P.R.C.).

## Conflict of Interest

The authors declare that the research was conducted in the absence of any commercial or financial relationships that could be construed as a potential conflict of interest.
